# Mono- and Dicationic DABCO/Quinuclidine Composed Nanomaterials for the Loading of Steroidal Drug: 3^2^ Factorial Design and Physicochemical Characterization

**DOI:** 10.3390/nano11102758

**Published:** 2021-10-18

**Authors:** Ana R. Fernandes, Elena Sanchez-Lopez, Antonello Santini, Tiago dos Santos, Maria L. Garcia, Amélia M. Silva, Eliana B. Souto

**Affiliations:** 1 Department of Pharmaceutical Technology, Faculty of Pharmacy, University of Coimbra, Pólo das Ciências da Saúde, Azinhaga de Santa Comba, 3000-548 Coimbra, Portugal; anaritavfernandes@gmail.com; 2 i3s—Instituto de Investigação e Inovação em Saúde, Universidade do Porto, R. Alfredo Allen 208, 4200-135 Porto, Portugal; tiago.f.santos@ineb.up.pt; 3 INEB—Instituto de Engenharia Biomédica, Universidade do Porto, Alfredo Allen 208, 4200-135 Porto, Portugal; 4 Faculdade de Engenharia, Universidade do Porto, R. Dr. Roberto Frias, 4200-465 Porto, Portugal; 5 Department of Pharmacy, Pharmaceutical Technology and Physical Chemistry, Faculty of Pharmacy, University of Barcelona, 08028 Barcelona, Spain; esanchezlopez@ub.edu (E.S.-L.); marisagarcia@ub.edu (M.L.G.); 6 Institute of Nanoscience and Nanotechnology (IN2UB), University of Barcelona, 08028 Barcelona, Spain; 7 Department of Pharmacy, Università di Napoli Federico II, Via D. Montesano 49, 80131 Napoli, Italy; asantini@unina.it; 8 Department of Biology and Environment, University of Trás-os-Montes e Alto Douro, UTAD, Quinta de Prados, 5001-801 Vila Real, Portugal; 9 Centre for Research and Technology of Agro-Environmental and Biological Sciences, CITAB, UTAD, Quinta de Prados, 5001-801 Vila Real, Portugal; 10 CEB—Centre of Biological Engineering, University of Minho, Campus de Gualtar, 4710-057 Braga, Portugal

**Keywords:** cationic nanoemulsions, DABCO surfactants, quinuclidine surfactants, ocular administration, triamcinolone acetonide

## Abstract

Oil-in-water nanoemulsions (NEs) are considered a suitable nanotechnological approach to improve the eye-related bioavailability of lipophilic drugs. The potential of cationic NEs is prominent due to the electrostatic interaction that occurs between the positively charged droplets with the negatively charged mucins present in the tear film. This interaction offers prolonged NEs residence at the ocular surface, increasing the drug absorption. Triamcinolone acetonide (TA) is one of the first pharmacologic strategies applied as an intravitreal injection in the treatment of age-related macular degeneration (AMD). Newly synthesized quaternary derivatives of 1,4-diazabicyclo[2.2.2]octane (DABCO) and quinuclidine surfactants have been screened with the purpose to select the best compound to formulate long-term stable NEs that combine the best physicochemical properties for the loading of TA intended for ocular administration.

## 1. Introduction

Oil-in-water nanoemulsions (NEs) are one of the methodologies for preparing formulations to improve the ocular bioavailability of lipophilic drugs [1,2]. Among NEs, cationic NEs present prominent potential due to the electrostatic interactions occurring between the positively charged eye-droplets with the negatively charged mucins expressed at ocular surface epithelia and are responsible for the tear film maintenance [3,4]. This electrostatic interaction offers prolonged NEs residence time at the ocular surface increasing the drug absorption [5]. Topical instillations of eye-drops are the common treatment for ophthalmic diseases. However, in the treatment of age-related macular degeneration (AMD), one of the first pharmacologic drugs, triamcinolone acetonide (TA), is only applied as intravitreal injections. TA presents important effects in the stabilization of the blood-retinal barrier and in the management of inflammation, and also exhibits relevant antiangiogenic and anti-fibrotic properties [6]. TA is a synthetic corticosteroid that, besides being well tolerated by ocular tissues, remains pharmacologically active for months after intravitreal injection, and is thus used in the treatment of several ocular diseases [6,7,8]. TA is also used to treat skin inflammatory diseases, and its formulation in transfersomes demonstrated a prolonged anti-inflammatory action in comparison to conventional formulations [9]. Conventional eye-drops are well accepted by patients; nevertheless, these formulations have some technical issues, namely, stability, solubility and low bioavailability (due to small residence time at the ocular surfaces) leading to a loss of about 90% of the administered drug as a result of blinking and solution drainage [8,10,11]. Ocular bioavailability of lipophilic drugs, such as TA, can be improved by formulating them in oil-in-water (*o*/*w*) NEs, in which the drug is solubilized at the innermost oil phase or at the *o*/*w* interface of the NEs [12]. The NE oil droplets have a nanometric size which leads to a large surface area that is an advantage to the drug diffusion and absorption [13]. The blinking and the composition of the tear are responsible for the NEs breakdown after administration. So, after NEs breakdown, the drug molecules are released and the oily phase of the NEs mixes with the tear film lipid layer [5]. Other advantages of NEs include enhanced ocular retention associated with extended effect duration, sustained drug-release, reduced systemic side-effects and, as NEs are able to interact with the tear film lipid layer, they can stay for a longer time in the conjunctival sac, acting as a drug depot [14,15,16]. Additionally, the NE droplet surface can be functionalized with cationic lipids or surfactants or polymers to form positively charged droplets, enhancing residence time [5]. 

**Figure 1:**
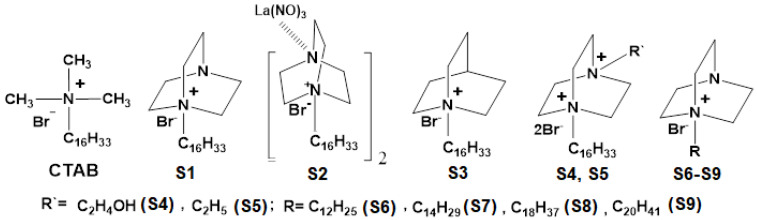
Structures of cetyltrimethylammonium bromide (CTAB), and quaternary derivatives of quinuclidine (S3) and of DABCO derivatives (S1, S2 and S4 to S9 surfactants) (Adapted from Ref. [17]).

Among the wide range of environmentally friend, cleavable, less toxic cationic and green cationic surfactants containing natural moiety of particular interest are the quaternary derivatives of 1,4-diazabicyclo[2.2.2]octane (DABCO; C_6_H_12_N_2_) and of 1-azabicyclo[2.2.2]octane (quinuclidine; C_7_H_13_N) due to their widespread applications in biotechnology and simple design [17]. The saturated bicyclic framework of these compounds is found in natural physiologically active compounds [17]. In addition, these surfactants display antibacterial [18] and antiviral [18] activity as well as improved solubility properties for hydrophobic dyes and drugs [17,19]. In this work, cationic NEs composed of mono- and dicationic DABCO and quinuclidine surfactants produced and supplied by Arbuzov Institute of Organic and Physical Chemistry of the FRC Kazan Scientific Center of Russian Academy of Sciences (S1–S9) (Figure 1) have been used to exploit their antimicrobial profile in drug delivery systems for ocular administration.

The choice of oils, surfactants, cationic lipids, polymers, isotonizing agents that are ophthalmically acceptable, is one of the most important steps to obtain a successful development of a stable and functional NE. The challenge is to have the potential to prolong the precorneal residence time of the formulation and then improve the bioavailability and, at the same time, it is essential to have an NE that is well tolerated, comfortable for patients and without non-irritant consequences. In this way, to achieve the optimal formulations for ocular delivery of TA, it is crucial to make a deep physicochemical characterization, i.e., measurement of droplets size, zeta potential, pH, osmolality, surface tension and viscosity. The aim of this work was the development of a cationic NE with new surfactants to load TA for ocular administration. Therefore, physicochemical characterizations were performed to choose the lead NE formulations with the greatest potential for further biopharmaceutical and tolerability studies.

## 2. Materials and Methods

### 2.1. Materials

Polysorbate 80 (Tween 80^®^) was purchased from Uniqema (Everberg, Belgium). Soybean oil, CTAB (cetyltrimethylammonium bromide), glycerol and triamcinolone acetonide (TA) were purchased from Sigma-Aldrich (Steinheim, Germany). Poloxamer 188 (Kolliphor 188) was purchased from BASF Schweiz AG (Kaisten, Germany). Cationic surfactants (mono- and dicationic DABCO and quinuclidine) were synthesized at the Arbuzov Institute of Organic and Physical Chemistry of the FRC Kazan Scientific Center of Russian Academy of Sciences (Kazan, Russia) [17,18,19]. Ultra-purified water was obtained from Milli^®^ Q Plus system (Darmstadt, Germany), home supplied.

### 2.2. Methods

#### 2.2.1. Factorial Design

The influence on the final properties of the NEs (mean particle size, polydispersity index (PI) and zeta potential (ZP)), of the soybean oil concentration (internal phase) and glycerol concentration (osmotic agent), as well as the amplitude of sonication, was evaluated by using a 3^2^ factorial design. This factorial design was composed of 3 variables that were set at 2-levels each. For each variable, we studied the lower and higher values that were represented by −1 and +1, respectively. The replication of the central point, represented by 0, was made three times in order to estimate the experimental error. The values of each level were chosen based on literature research [13]. The NEs were produced, and the data were analyzed using STATISTICA 7.0^®^ (Statsoft, Inc., Tulsa, OK, USA) software. An analysis of variance statistical test, ANOVA, was performed for each parameter to be able to identify the implication of the effects and the interactions between them in the final NEs. A *p*-value < 0.05 was considered statistically significant.

#### 2.2.2. Preparation of Nanoemulsions

NEs were produced by dispersing the oil phase (composed of soybean oil, tween 80 and a cationic surfactant), heated at the same temperature, in an aqueous solution (composed of glycerol, poloxamer 188 and water) using a probe sonication Qsonica 4435 Q55 Sonicator Microprobe, 1/4”, with 0.635 cm of tip diameter (Sonics Vibracell, Newtown, CT, USA). The composition of each developed formulation is indicated in Table 1. Two different amplitudes of sonication were studied, i.e., 60 or 100% of power output. Each formulation studied was produced with a final volume of 30 mL. Briefly, both oil and aqueous phases were heated up (~50 °C) in a water bath. The oil phase was dispersed in the aqueous phase and was processed using a sonication probe for 5 min. After this, the emulsion was transferred to an ice bath. The pre-formulation studies were performed using cetyltrimethylammonium bromide (CTAB) (at 50 μg/mL, Table 1) as surfactant model, once CTAB is a typical cationic surfactant [20]. The independent variables were defined as: percentage of soybean oil, percentage of glycerol and the amplitude of the sonication and dependent variables as: size, polydispersity index (PI) and zeta potential (ZP). Using STATISTICA 7.0^®^ software a 3^2^ factorial design was implemented and 11 formulations, to achieve the optimal formulation (Table 2), were made. After the pre-formulation studies using CTAB as the model surfactant, this cationic lipid was replaced by the synthesized cationic surfactants (Figure 1) using their respective critical micelle concentration (CMC), to produce nine formulations [17].

#### 2.2.3. Physicochemical Characterization

Dynamic light scattering (DLS) was the method used to analyze the particle size and polydispersity index (PI). This method records, in the microsecond time scale, the scattered light intensity variation. In the DLS method, the particles in gas or liquid were subjected to Brownian motions, and their movement can be perfectly described using the Stokes-Einstein equation. In this study, NEs size and PI were determined in triplicate using the Zetasizer Nano ZS (Malvern, UK). Values are presented as the mean of triplicate runs per sample. For each measurement, the NE was diluted in Milli-Q water to an appropriate concentration to avoid multiple scattering. The ZP is normally determined as the potential difference between the medium of the dispersion and the fluid attached to the dispersed particle. ZP measurements were performed by electrophoretic light scattering using a Zetasizer Nano ZS (Malvern, UK). For analysis, samples were placed in a Flow Cell at 25 °C, diluted with Milli-Q water to a proper concentration. ZP was calculated using the Helmholtz-Smoluchowski equation that was incorporated in the software system. Results are presented as the mean of triplicate runs per sample.

#### 2.2.4. Accelerated Stability Analysis

LUMiSizer^®^ (Boulder, CO, USA) is a dispersion analyzer commonly used in the characterization of substances separation in a mixture, i.e., sedimentation, creaming, flotation or consolidation. The simulated long-term physical stability of NE, without prior dilution, was assessed by placing 1 mL of each sample in rectangular test-tubes (2 mm optical path) and then subjected to rotor speed of 4000 rpm (centrifugal force 2300× *g*) at 25 °C, as described in [21], a total of 850 profiles were obtained in intervals of 30 s. These assays permitted differentiating between several mechanisms of instability at an accelerated and known rate. Results were analyzed, using the SEPView^®^ software (LUM GmbH, Berlin Germany). The instability index was calculated by the software using the clarification at each separation time divided by the maximum clarification [22]. The transmission profiles are the result of the variation of transmitted light over time and space and give us the information about the kinetics of the separation process and the velocity of migration of particles (which is related to the particle size) [23,24].

#### 2.2.5. Encapsulation Efficiency

In order to determine the efficiency of encapsulation, the NEs were submitted at centrifugation with Amicon^®^ Ultra Centrifugal Filters Ultracel (Millipore, Darmstadt, Germany) for 15 min at 13,400× *g* to isolate the particles out of the suspension. The free-TA was measured by the indirect method. The supernatant was measured using a plate reader to determine the drug concentration. The standards of the calibration curve were prepared by diluting the TA in Milli-Q water with 20% of ethanol to assure the total dilution. The concentration of each standard of the calibration curve and the concentration of the supernatant were measured in a BioTek Synergy HT plate reader at 240 nm (BioTek Instruments, Winooski, VT, USA). The encapsulation efficiency (*EE*) of TA in NEs was calculated as follows:

(1) $$EE\%=\frac{W_{TA}-W_{S}}{W_{TA}}\times 100$$

where *W_TA_* is the mass of triamcinolone acetonide (TA) used for the production of the loaded NEs and *W_s_* is the mass of TA quantified in the supernatant. Centrifugal filter units were used with a cut-off of 50 kDa, i.e., 50,000 nominal molecular weight limits (NMWL).

#### 2.2.6. Surface Tension

The surface tension was assessed using KSV Sigma 70 Force Tensiometer (Helsinki, Finland) that applies the Du Noüy ring detachment method taking into account the correction of Huh and Mason for interface distortion. The surface tension of NEs was measured 5 times at 37.3 °C, as described in [25].

#### 2.2.7. Osmolality Assessment

The values of osmolality of the NEs were obtained using the EquipWescor Vapor Pressure Osmometer VAPRO (Model 5520) (Logan, UT, USA). A 10 microliter of each sample was placed into a solute-free paper disc in the sample holder. The measurement initiates when the sample holder is pushed into the equipment. The measurement cycle takes 80 s [25]. The results of Vapro displays in Standard International units, mOsm/kg.

#### 2.2.8. Rheological Behavior

The rheology studies were performed on a rheometer Rheo Stress RS 100 (Haake Instruments, Karlsruhe, Germany), applying the frequency sweep test. An oscillation frequency sweep test was applied over a frequency range from 0 to 10 Hz. The storage modulus (G′), loss modulus (G″) and the complex viscosity (η^*^) of NE were determined as a function of the frequency at constant stress amplitude of 5 Pa (linear viscoelastic region). All experiments were performed at room temperature.

#### 2.2.9. Stability of NEs in Simulated Tears

The stability of the formulations was tested in commercialized eye cleaning solutions to anticipate whether droplets aggregation occurs upon eye administration. Two different dilutions of NEs were tested on these solutions and the modifications on the physicochemical properties were studied. One solution is saline solution sterile (sterile sodium chloride 0.9%) and another solution is a buffer with phosphates sterile and pH neutral (phosphate solution 4.9%), normally used to neutralize acids and alkaline substances (neutral solution).

#### 2.2.10. Morphology Analysis TEM

NEs were analyzed by transmission electronic microscopy (TEM). Samples were mounted on a grid without staining and, after drying at room temperature, were examined using the equipment Tecnai^TM^ G^2^ Spirit BioTWIN (FEI Company, Hillsboro, OR, USA). Image J Software (Version 1.47) (Bethesda, MD, USA) was used to analyze and measure the NEs samples.

## 3. Results and Discussion

This work aimed to develop NEs able to deliver TA after topical administration in the eye to treat or prevent AMD or other inflammatory and angiogenic ocular diseases. The components of NEs altogether contributed to achieve long-term stable NEs for the delivery of the poorly-water soluble TA. Soybean oil has been selected as a component of the inner phase of NEs because it is a recognized non-irritating and biocompatible pharmaceutical excipient [26]. Cationic quaternary ammonium surfactants (i.e., mono- and dicationic DABCO and quinuclidine) are act as preservatives and contribute for the electrostatic stabilization of the droplets due to the cationic charge at the interface [13]. A combination of one cationic surfactant with a non-ionic surfactant (Tween 80) was used. Tween 80 is described as a harmless, hydrophilic nonionic surfactant, and can cause reversible changes in the permeability of the ocular surface. This non-ionic surfactant is used as a lubricant in eye drops, promoting stereochemical stabilization of the inner oil droplets of the NEs and contributes also with antimicrobial properties [27]. The use of cationic surfactants in combination with non-ionic surfactants has already been recommended to improve colloidal stability [28]. Poloxamer 188 (a non-ionic emulsifier) was used as a co-emulsifier to reduce the size distribution [29]. Conjugation of tween 80 and poloxamer 188 is reported to improve the spreading over the entire cornea-conjunctiva surface [30].

A factorial design of a new pharmaceutical formulation requires the identification of the influencing parameters that will affect significantly the final product. The experimental factorial design aims to study the effect of the different independent variables on the final properties of the new pharmaceutical formulation. Factorial design is a statistical analysis that provides a way to select the most optimal experimental conditions for the new pharmaceutical formulation. Those conditions are, for example, different ratios of surfactants, different concentrations of lipids, different conditions of production, i.e., different velocities of sonication. This statistical analysis also estimates the influence of independent variables on results of the dependent variables, i.e., mean particle size, polydispersity index (PI) and zeta potential (ZP). In this study, these dependent variables were studied to determine the physicochemical properties of the NEs. A factorial design study was performed to maximize the experimental efficiency using a minimum of experiments to obtain the optimal NEs.

The challenge of the experimental design is the agreement with increasing number of the factors and levels. The factorial design was composed of three variables that were set at two-levels each (3^2^). In this case, 11 formulations were made with different concentrations of glycerol and soybean oil, unchanged concentration of CTAB (50 μg/mL) and different amplitude of sonication to achieve the optimal formulation (Table 2). CTAB has been selected as a cationic lipid/surfactant as it has been commonly used in the production of cationic nanoparticles for ocular administration at a non-cytotoxic concentration [20,31]. NEs were stored at 4 °C. The mean particle size, the polydispersity index and the ZP were measured on the day of production. The obtained results are shown in Table 3.

Table 2 shows the amplitude used for each formulation as well as the concentration of each lipid. The other constituents of the formulations that are not in this table are unaltered, i.e., their concentrations are fixed. The column ‘pattern’ identifies the lower and higher values represented by − that means −1 and + that means +1, respectively. The pattern 0 represents the central point that is the intermediate value of the variables. The ‘0’ was represented in Table 2 three times because was made three formulations with these values in order to estimate the experimental error of the assay. The results for the dependent variables were described in Table 3. For every three dependent variables, analysis of the variance (ANOVA) was performed using a confidence level of 95% confidence interval (*p*-value = 0.05).

**Figure 2:**
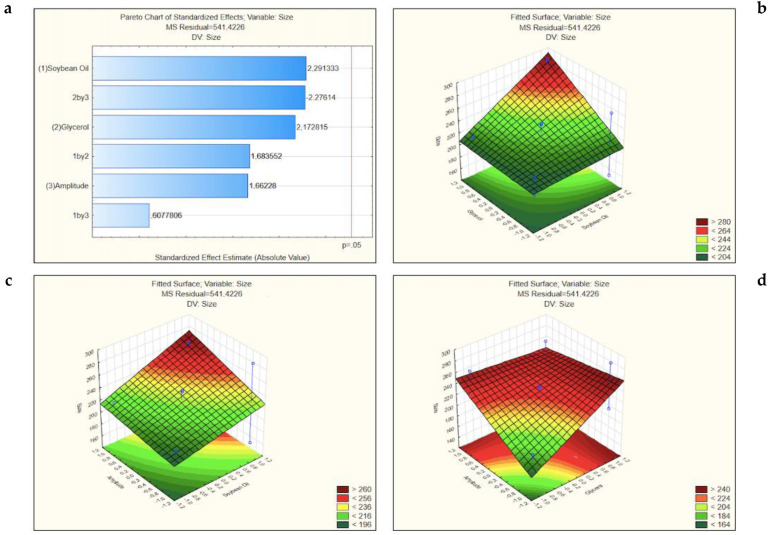
Pareto chart (**a**) and surface response graphs of the influence of the concentration of the glycerol and soybean oil (**b**), of soybean oil and amplitude (**c**) and of glycerol and amplitude (**d**) on the particle size.

The obtained results were used to build the Pareto charts and the fitted surface graphs for the different dependent variables. The response coefficients for the dependent variables were studied for their statistical significance and the results are shown in Figure 2. The *t*-value of effects are set on the Pareto chart. The variation of the low value to a high value of the soybean oil concentration had a positive effect on the particle size, i.e., *t*-value = 2.291333 (Figure 2a). Similarly, the interaction between the variation of the soybean oil and glycerol from the lower to higher values had a positive effect on the particle size, i.e., *t*-value = 1.683552. Likewise, the variation of amplitude from lower to higher values had a positive effect on the particle size, i.e., *t*-value = 1.66228. The same happened in the values of glycerol, i.e., *t*-value = 2.172815. As well as the interaction between the variation of the soybean oil and the amplitude from the lower to higher values, *t*-value = 0.6077806. If the *t*-value of effects set on the Pareto charts is less or equal to the significant level (*p* < 0.05), this reveals that there is a statistically significant association between the response variable and the term and statistical significance, meaning that there is a good chance that we are right in finding that a relationship exists between two variables. As it is possible to see in the Pareto charts, all the *t*-values are less than *p* = 0.05. On the other hand, the interaction between the variation of the glycerol and amplitude from lower to higher values had a negative effect on the particle size (*t*-value = −2.27614).

The interactive effects between the different dependent variables studied were plotted in three-dimensional response surface graphs (Figure 2). In these surface response charts, the variations in the response values are in the *Z*-axis against the levels of the three independent variables (glycerol in *X*-axis and soybean oil in *Y*-axis in the first graph, amplitude *X*-axis and soybean oil in *Y*-axis in the second graph, amplitude in the *X*-axis and glycerol in *Y*-axis in the last graph). The combination of high concentrations of glycerol and soybean oil, as well as the combination of the high concentrations of soybean oil and the amplitude increases the particle size to values above 300 nm while the best results to the size particle are obtained when the combination of medium or low amplitude of sonication with medium or lower concentration of glycerol. If in this last combination, any variable has the higher value studied, the size particle achieves easily more than 250 nm.

**Figure 3:**
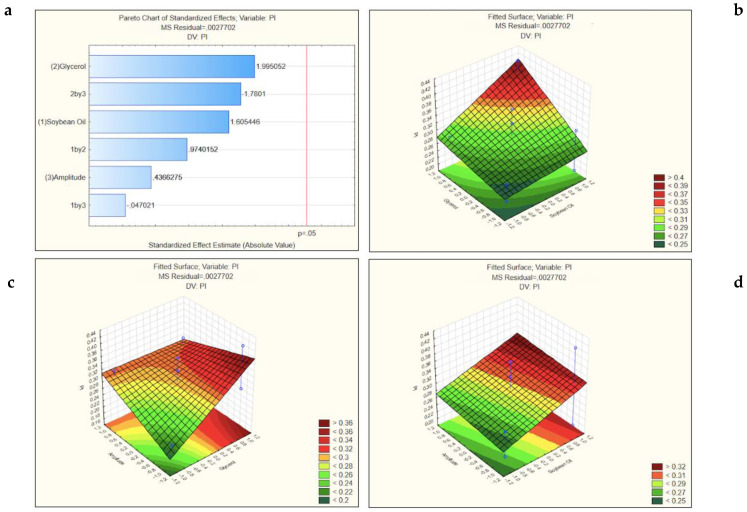
Pareto chart (**a**) and surface response graphs of the influence of the concentration of the glycerol and soybean oil (**b**), of soybean oil and amplitude (**c**) and of glycerol and amplitude (**d**) on the polydispersity index (PI).

A higher concentration of lipids leads to an increase of the viscosity of the formulations, which promotes the particle agglomeration and then affects the mean particle size [32]. The results of the polydispersity index (PI) (Figure 3) were also statistically significant. The *t*-value obtained from the variation of the low value to a high value of the concentrations of glycerol and soybean oil and the amplitude (1.995052, 1.605445 and 0.4366275, respectively) had a positive effect on the PI. The same was seen for the interaction between the concentration of glycerol and soybean oil, 0.9740152. Differently, the *t*-value for the variation of the low values to high values of the interaction of concentration of glycerol and the amplitude showed a negative effect on the PI, −1.7801. Likewise, the interaction between the soybean oil and the amplitude, −0.047021.

**Figure 4:**
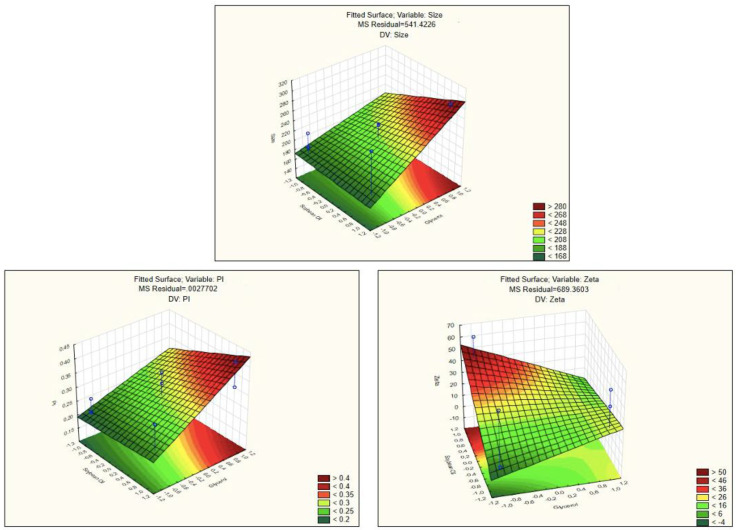
Surface response graphs of the influence of the concentration of glycerol and soybean oil with the lowest amplitude studied (−1, i.e., 60) on the mean size (**upper**), polydispersity index (**lower left**) and on the zeta potential (**lower right**).

Based on these findings, the amplitude that was selected as optimal to produce the formulations and to proceed to the in vitro studies was the lower, 60. The three-dimensional response surface graphs for the size and for the PI both showed that in all the interactions of the amplitude with the different concentrations of the surfactants, the lowest value studied for the amplitude present the smallest particle size and origin particles more homogeneous due to the lowest PI. With these results, we decided to use de minor amplitude (−1) in the next assays. According to this, the following surface response graphs (Figure 4) represents that condition, i.e., with the amplitude value of 60 what is the influence of different concentrations of soybean oil and glycerol in the mean size of the formulations, the PI and ZP. In all of the surface response graphs (Figure 3), increasing glycerol concentration creates NEs with higher mean size and with high values of PI and ZP values around zero. As known, the ZP (i.e., the electrical charge at the NEs surface) reflects the long-term physical stability and shows the tendency for particles aggregation. Higher ZP values, either positive or negative, mean that the formulations will have greater long-term stability and long shelf-life [33]. The particle aggregation after production is less expect to occur for charged particles with ZP > |20| mV, since there is electrostatic repulsion between particles with the same electrical charge [34]. The NEs produced using CTAB are thus reported to have higher stability according to the ZP, these are the NEs represented in the Table 3 by the pattern −++, −−+, +−−, −+− (nanoemulsions 2, 3, 4 and 5, respectively).

In addition to the surface response study described above, we used the LUMiSizer^®^ to analyze the shelf-life of the NEs using the space and time resolved extinction profiles. Based on these profiles, demixing processes were measured regarding the clarification velocity, the velocity of sedimentation and flotation of particles, the turbidity and separated phase components—liquid or solid. This equipment uses the centrifugal sedimentation approach to estimate the shelf-life of formulations at their original concentration and a fast stability ranking. These estimations take minutes/hours instead of days/months/years. The evolution of the transmission profiles along the time facilitates the analysis of their demixing behavior of tested formulations and their stability. It is possible to extrapolate results to estimate the dispersion shelf-life of undiluted dispersions in minutes instead of months or years [33]. Stable colloidal dispersions depict the formation of a flatbed under a centrifugal field, while the aggregated particles usually show a step-profile [35]. Then, the centrifugal accelerations cause different sedimentation profiles and velocities of formulations with heterogeneous size ranges. The instability phenomenon is related to changes in the particle size distribution, due to their interaction, and to migration particles [33]. As seen in Table 4, the formulation that showed a lower instability index was the nanoemulsion 4 (0.214).

**Figure 5:**
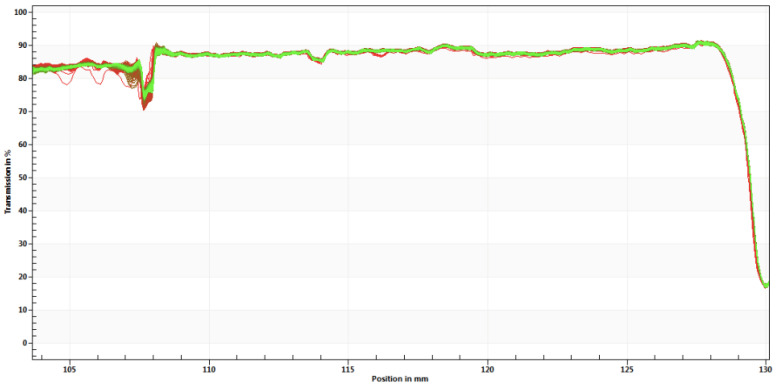
Instability profile of nanoemulsion 4 on the day of production (day 0).

According to this approach, the most stable formulation was nanoemulsion 4 (+−−). This result is in agreement with the surfaces responses obtained previously where the minor amplitude and concentration of glycerol give us better results either in mean size, PI and ZP values. The transmission profile of NE 4 is shown in Figure 5.

**Figure 6:**
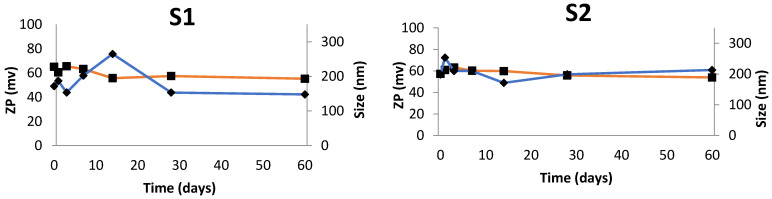

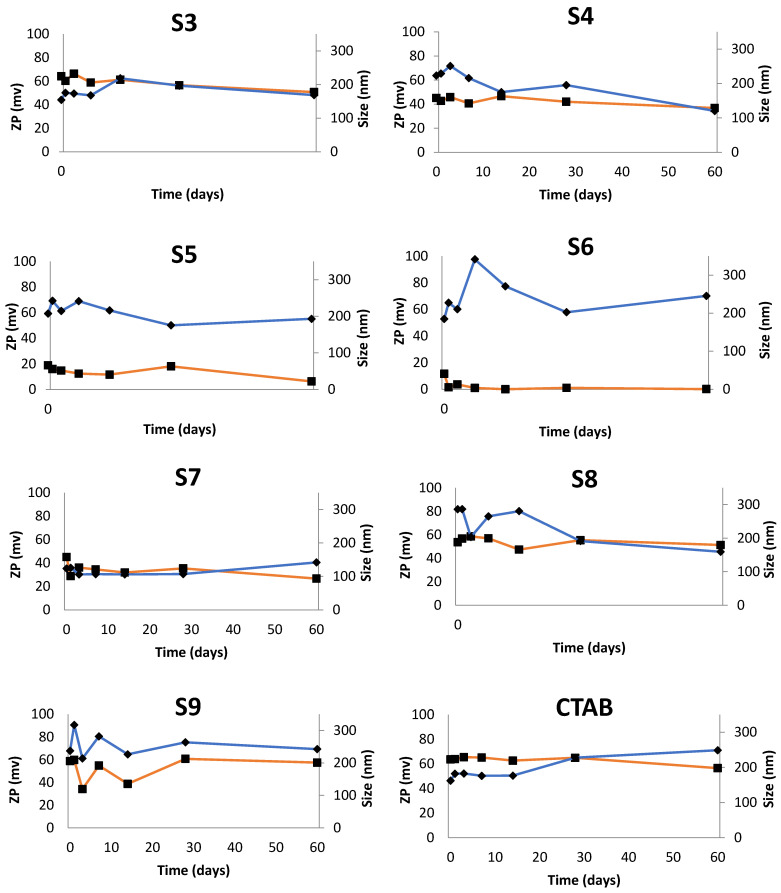
Mean size and zeta potential (ZP) monitored over a period of 60 days of NEs produced with surfactant 1 to 9 (S1 to S9) and with CTAB (used as model surfactant). Blue line represents the mean size and orange line the ZP.

The instability profile of NE4 showed a very high level of clarification since the beginning of the assay, which demonstrates that no migration or sedimentation occurred. After these preliminary studies, nine NEs were produced replacing CTAB with the synthesized surfactants (as shown in Figure 1) using the composition of nanoemulsion 4 selected as the optimal combination. All the formulations were monitored for two months taking into account the mean size and the ZP. The results are shown in Figure 6.

The most stable NEs were found to be those obtained with S2 and S7 (Figure 6). These NEs depict similar mean size and ZP profiles during the assay. Huge variations in size and ZP were recorded over time for the remaining surfactant-based NEs. The PI of NEs produced with S2 and S7 was maintained around 0.21–0.23 over the 60 days, which ensures that samples are able to keep the same physicochemical properties over time.

Osmolality translates the total concentration of solute in a solution, i.e., formulations with a low solute concentration have a low osmolality and formulations with a high concentration of solutes have a high osmolality value [36]. In the case of ocular delivery of drugs, formulations should not cause any discomfort upon administration, thus tolerability of the formulations, pH and osmolality should be considered. The cornea reacts upon changes in pH and osmolality, which can provoke reflex blinking and tearing. The osmolality of the NEs produced with the nine surfactants and CTAB was studied over a period of 60 days, stored at 4 °C. Table 5 shows the obtained results. All the formulations have a hypotonic profile as the osmolality was lower than the physiologic values (approximately 289 mOsm/kg), which promotes fluid absorption. The use of hypotonic solutions is highly recommended in, for example, dry eye syndrome due to higher values of tear osmolality in this disease. There are no adverse effects reported upon the use of hypotonic solutions [37,38].

**Figure 7:**
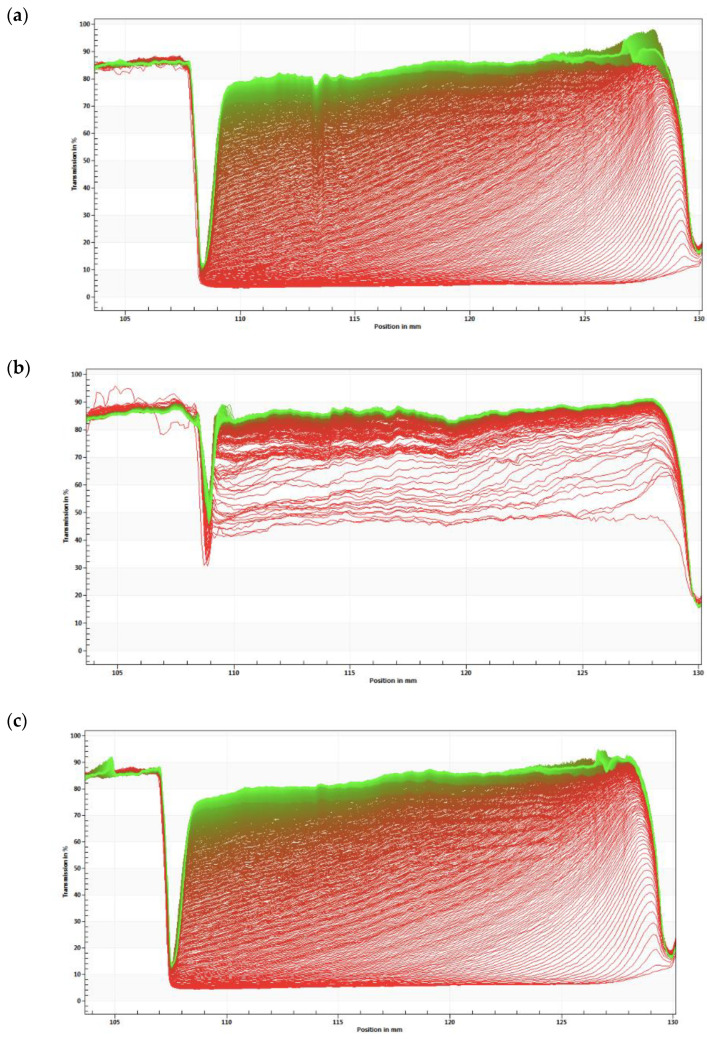
Instability profile of S2-based NE (**a**), S7-based NE (**b**) and CTAB-based nanoemulsion (**c**).

These S2- and S7-based NEs were then selected for further studies. Figure 7 compares the instability profiles obtained for S2, S7 and CTAB. Figure 7a,c shows profiles of samples with a regular mean size distribution. These profiles are suggested by the symmetrical spacing observed for the majority of the profile. Figure 7b shows an almost constant profile over time. This profile presents the most homogeneous formulation, i.e., it shows higher stability compared to S2 and CTAB. After this, and taking into account the stability of the formulation, surfactants S2 and S7 were chosen for further studies. These formulations were then produced to carry with the TA.

**Figure 8:**
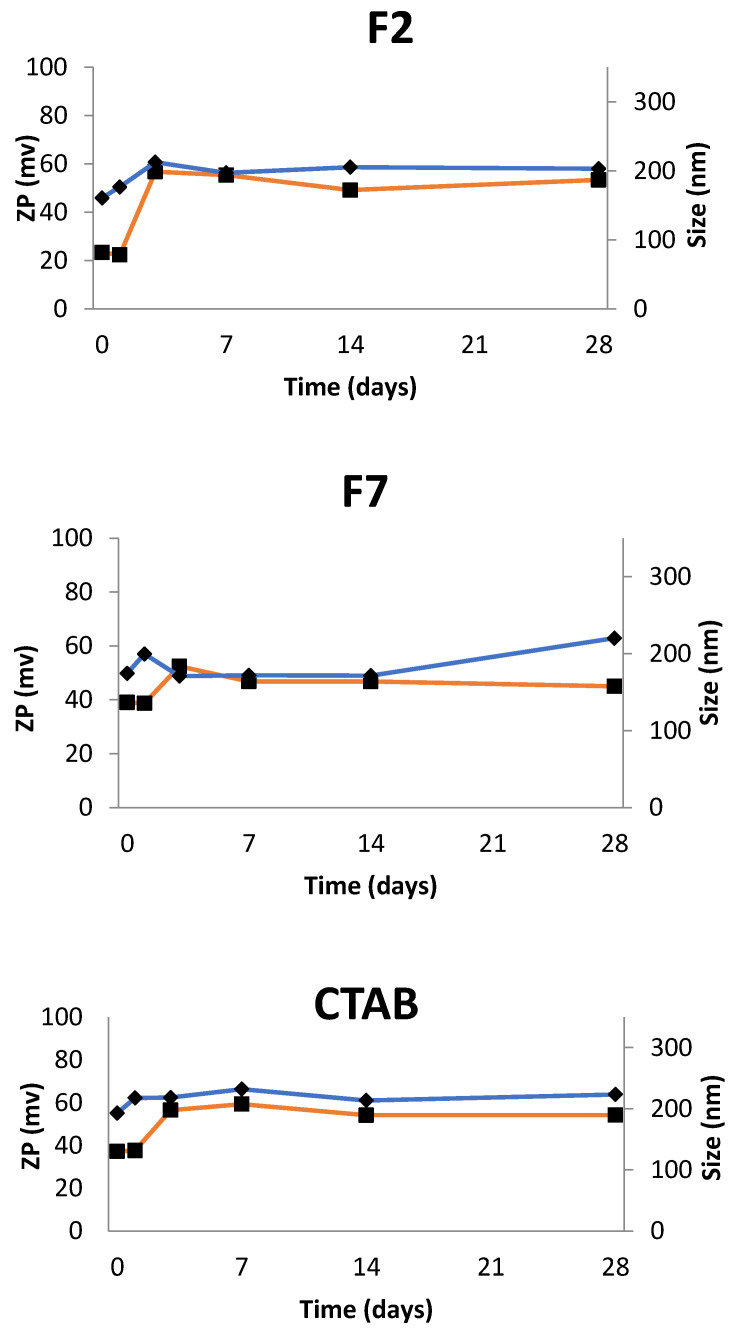
Mean size and zeta potential (ZP) recorded for the formulations F2, F7, CTAB-based nanoemulsion containing TA, over a period of 28 days.

Freshly prepared TA-loaded NEs (TA at 0.005%), the mean particle size and zeta potential of samples were monitored for 28 days (Figure 8). Formulation 2 (F2) depicted mean size as the nanoemulsion with the same composition but without TA (S2). In terms of ZP, in the F2 there was an increase of the values in the first three days after production and then stabilized to the same values that the S2. In the case of Formulation 7 (F7), the mean size and ZP showed stable values over time. These values were significantly higher when compared to the S7. For CTAB-based nanoemulsion containing TA, the mean size values are more consistent over the same period of time. ZP values for this formulation stabilized three days after production, as well as in F2 and F7.

The encapsulation efficiency was measured indirectly, by determining the amount of free drug in the supernatant obtained by centrifugation (Table 6). Formulation F7 shows the highest encapsulation efficiency for TA, whereas CTAB-based formulation depicted the lowest encapsulation efficiency. For these three formulations, the variation of osmolality was studied over a period of 28 days and the results are shown in Table 7.

The surface tension, a fundamental property of liquids surface, is defined as the energy, or work, required to increase the liquid surface area due to intermolecular forces. Surface tension is described as the ability to a surface of a portion of liquid be attracted by another surface or portion of liquid [39]. Higher surface tension results from stronger interaction between the molecules of the liquids’ surface with the neighboring molecules. As temperature decreases the surface tension increases due to more intermolecular bounds [39]. The surface tension was obtained using KSV Sigma 70 equipment. The NEs were measured five times at 37.3 °C. For F2 the surface tension was 24.32 ± 0.13 mN/m. In the case of F7, the surface tension was 24.62 ± 0.05 mN/m. In the formulation prepared with CTAB, the surface tension was 21.55 ± 0.29 mN/m. It should be taken into account that the tolerability limits of osmolality for ophthalmic formulations range from 171 to 1711 mOsm/kg. Most of the commercialized ophthalmic products, such as lubricants, have osmolality around 150–250 mOsm/kg [40]. F2 is the nanoemulsion depicting an osmolality value according to the reference. The use of hypotonic formulations, such as F2, is required to decrease the tear osmolarity from abnormally high values [41].

**Figure 9:**
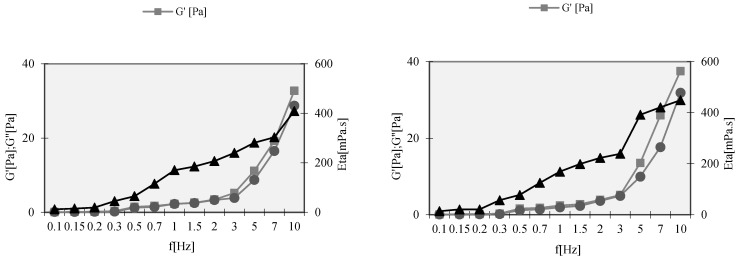
Rheology studies of formulation F2 (**left**) and formulation F7 (**right**).

The rheology studies were performed using the frequency sweep test (Figure 9). An oscillation frequency sweep test was applied over a frequency range from 0 to 10 Hz. The storage modulus (G′), loss modulus (G″) and the complex viscosity (η^*^) of NEs were determined as a function of the frequency at constant stress amplitude of 5 Pa (linear viscoelastic region). The G′ can be used as a measure of the elastic component of the sample and the G″ as the viscous component. In both formulations, F2 and F7, the elastic modulus (G′) is dominant over the viscous modulus (G″) and both of these are dependent on frequency. The profiles of both formulations showed similar behavior in function of frequency.

**Figure 10:**
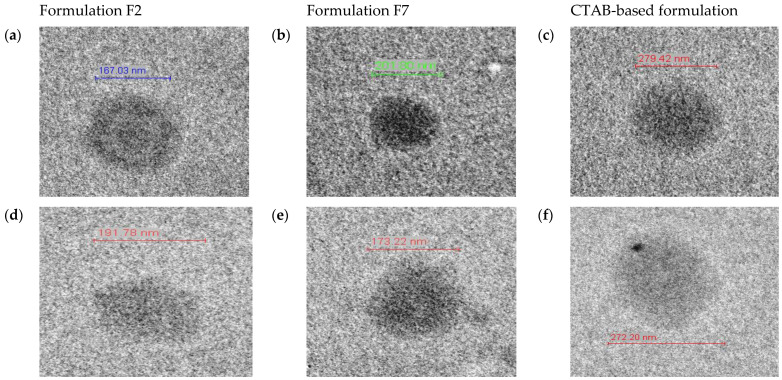
Images obtained using a transmission electron microscopy. F2 at day 0 (**a**) and at 28 days after production (**d**). F7 at day 0 (**b**) and at day 28 days after production (**e**). CTAB at day 0 (**c**) and at day 28 after production (**f**).

The analysis by transmission electron microscopy (TEM) is shown in Figure 10. The a–c images were obtained on the day of production and d–f were obtained 28 days after production. During this time, the samples were kept at 4 °C. NE droplets are clearly visible, and the droplet size analysis was performed by the software of the Tecnai^TM^ G^2^ Spirit BioTWIN microscopic. The shape of the droplets was spherical in all formulations and the appearance maintained similar after 28 days of production (Figure 10d–f).

**Figure 11:**
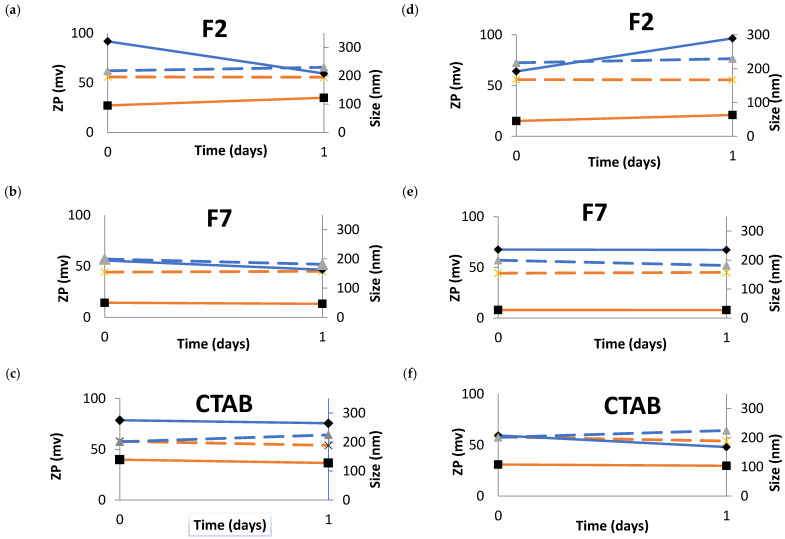
Zeta potential variation recorded on day zero and 24 h after production of NEs, determined in sterile saline solution, for two distinct dilutions at 1:1 (left-hand side panels, (**a**–**c**)) and at 1:3 (right-hand side panels, (**d**–**f**)). Blue line refers to: mean particle size of NEs after dilution in the different solutions (continuous line) and mean particle size of original NEs (dashed line); orange line refers to: ZP of NEs after dilution in the different solutions (continuous line) and ZP of original NEs (dashed line).

The stability of the NEs was tested using an ocular formulation commonly commercialized as a cleaning solution for the eyes. One solution is a sterile saline solution, and the other solution is a phosphate sterile buffer at neutral pH, normally used to neutralize acids and alkaline substances (neutral solution). These NEs were physicochemically characterized during 24 h in terms of mean particle size and ZP in these fluids. The results obtained were compared with the original NEs (results in dashed line, Figure 11). In both dilutions, in all the NEs, the ZP was maintained almost the same during the assay. However, in all the profiles, the ZP of the NEs diluted in the sterile saline solution is significantly lower when compared to the original NE.

The more concentrated F2 (Figure 11a) shows initially an increase in the mean particle size when in contact with the saline solution comparing to the original NE; however, the mean particle size decreases dramatically to values similar to the original F2 NE. Whereas, for the more diluted F2 (Figure 11d) the behavior is exactly the opposite when in contact to the saline solution there was a decrease in the mean particle size and after this, there was an increase in the mean particle size to values ≈ 290 nm.

The F7/saline solution more concentrated (Figure 11b) did not show a significant difference in the mean particle size when compared to the F7 original NE during the assay. F7 more diluted (Figure 11e) in saline solution showed the same mean particle size in all the assay but this value is higher when compared to the original F7 NE.

**Figure 12:**
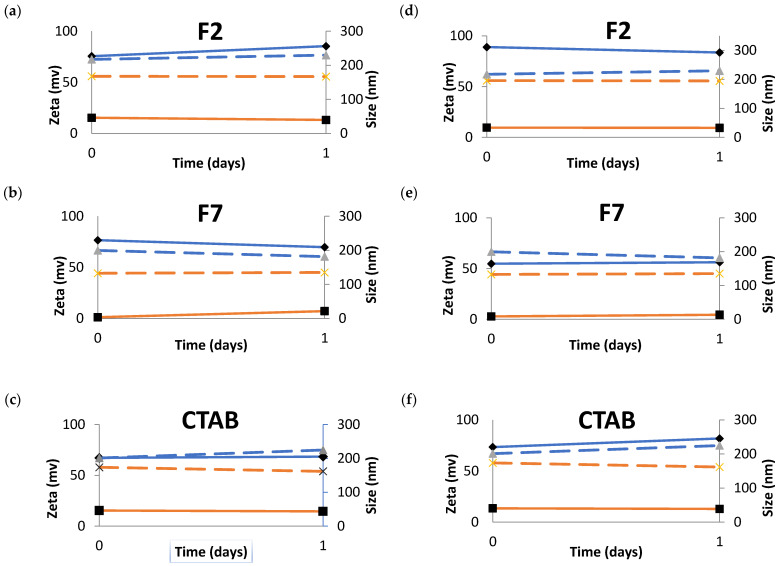
Behavior of NEs in sterile phosphate buffer at neutral pH for two different dilutions, at 1:1 (left-hand side panels, (**a**–**c**)) and at 1:3 (right-hand side panels, (**d**–**f**)). Blue line refers to: mean particle size of NE after dilution in the different solutions (continuous line) and mean particle size of original NEs (dashed line); orange line refers to: ZP of NEs after dilution in the different solutions (continuous line) and ZP of original NEs (dashed line).

The CTAB/saline solution more concentrated had the same mean particle size in all the assay, but this value is higher when compared to the original CTAB NE. For CTAB/saline solution more diluted, there was an increase if the mean particle size during the assay. In the beginning, the mean particle size was similar to the original CTAB NE and during the assay this value decreased significantly. The results for the dilution in the sterile phosphate buffer at neutral pH are shown in Figure 12.

Similar to the dilutions in the sterile saline solution, the ZP was maintained almost the same during the assay. Nevertheless, in all the profiles, the ZP of the NEs diluted in sterile phosphate buffer at neutral pH is significantly lower when compared to the original NE.

The F2 (dilution 1:1) (Figure 12a) initially shows an increase in the mean particle size when in contact with the sterile phosphate buffer at neutral pH comparing to the original F2 NE, and during the assay, the mean particle size continues to increase. The F2 more diluted (1:3) (Figure 12d) behavior is exactly the same in the beginning, i.e., increase the mean particle size when in contact with the sterile phosphate buffer at neutral pH and this value is maintained during the assay.

The F7/buffer with phosphates sterile at neutral pH at 1:1 dilution (Figure 12b) showed a slight increase in the mean particle size when compared to the F7 original NE during the assay. F7 more diluted (Figure 11e) in sterile phosphate buffer at neutral pH showed the same mean particle size in all the assay and this value is slightly lower than the reference.

The CTAB/buffer with phosphates sterile and pH neutral at 1:1 dilution had the same mean particle size and ZP in all the assay. For CTAB/buffer with phosphates sterile and pH neutral more diluted, there was an increase of the mean particle size during the assay. However, the ZP is the same during the 24 h. There was an enormous increase in the osmolality values of the NE when dissolved in the ocular formulation commonly commercialized as cleaning solutions of the eye (Table 8). The values of the osmolality in all the concentrations and in both solutions despite the increase were in the gap of the permissible osmolality values. The sterile saline solution has 279 mOsm/kg and the sterile buffer with phosphates and neutral pH has 704 mOsm/kg. As shown in Table 8, an increase in the osmolality was recorded upon dilution for the formulations. However, when compared to our developed NEs, the use of the saline solution and/or phosphate buffer does not contribute to improve the quality of the formulations for ocular administration; indeed, the increased osmolality seen with these commercial solutions, in comparison to our optimal formulations loaded with triamcinolone acetonide (Table 7), may induce a cytotoxic effect in the eye. Dutesco et al. showed that hypertonic formulations change the tear osmolarity and consequently induce ocular inflammation [37].

## 4. Conclusions

Newly synthesized quaternary derivatives of 1,4-diazabicyclo[2.2.2]octane (DABCO) and quinuclidine surfactants were the compounds used to formulate long-term stable nanoemulsions. Nanoemulsions revealed optimal physicochemical properties for the loading of triamcinolone acetonide intended for ocular administration. In the treatment of age-related macular degeneration, triamcinolone acetonide is one of the first pharmacologic drugs used that is only applied as intravitreal injections. Triamcinolone acetonide is a synthetic corticosteroid that is well tolerated by ocular tissues. The developed nanoemulsions loading triamcinolone acetonide showed long-term stability and physicochemical characteristics that are aligned with the requirements for ocular administration. Formulation F2 and formulation F7 presented a monodispersed population, i.e., higher stability comparing to the others and in the future, it will be recommended to conduct further studies in order to test their behavior in vitro and in vivo assays.

## Figures and Tables

**Table 1:** Composition of the developed nanoemulsions containing CTAB or a cationic Surfactant 1 to 9 (S1 to S9, for structure see Figure 1) (caption: CMC, critical micelle concentration).

	Soybean Oil (% *w*/*w*)	Tween 80 (% *w*/*w*)	Poloxamer 188 (% *w*/*w*)	Glycerol (% *w*/*w*)	Ctab (μg/mL)	Cationic Surfactants (CMC, in mM)
Pre-formulation	1.00 to 2.00	0.20	0.01	1.50 to 2.50	50	-
Formulation S1	2.00	0.20	0.01	1.50	-	1.00
Formulation S2	2.00	0.20	0.01	1.50	-	0.33
Formulation S3	2.00	0.20	0.01	1.50	-	0.80
Formulation S4	2.00	0.20	0.01	1.50	-	2.00
Formulation S5	2.00	0.20	0.01	1.50	-	3.00
Formulation S6	2.00	0.20	0.01	1.50	-	11.00
Formulation S7	2.00	0.20	0.01	1.50	-	4.00
Formulation S8	2.00	0.20	0.01	1.50	-	0.12
Formulation S9	2.00	0.20	0.01	1.50	-	0.08

**Table 2:** Experimental factorial design using CTAB as model surfactant.

Formulation	Pattern	Soybean Oil	Glycerol	Amplitude	Soybean Oil (g)	Glycerol (g)	Amplitude
1	000	0	0	0	0.45	0.60	80
2	−++	−1	1	1	0.30	0.75	100
3	−−+	−1	−1	1	0.30	0.45	100
4	+−−	1	−1	−1	0.60	0.45	60
5	−+−	−1	1	−1	0.30	0.75	60
6	+++	1	1	1	0.60	0.75	100
7	000	0	0	0	0.45	0.60	80
8	000	0	0	0	0.45	0.60	80
9	−−−	−1	−1	−1	0.30	0.45	60
10	++−	1	1	−1	0.60	0.75	60
11	+−+	1	−1	1	0.60	0.45	100

**Table 3:** Response dependent variables and pH and osmolality of the three independent factors presented in Table 2 for all the 11 produced NEs (Captions: z-Ave, mean particle size; PI, polydispersity index; ZP, zeta potential).

Nanoemulsion	Pattern	z-Ave (nm)	PI	ZP (mV)	pH	Osmolality (mOsm/kg)
1	000	240.2	0.369	0.032	4.019	188
2	−++	214.6	0.269	52.000	4.109	257
3	−−+	216.9	0.262	47.600	3.953	151
4	+−−	162.1	0.203	63.600	4.030	154
5	−+−	207.6	0.282	38.200	4.839	238
6	+++	262.9	0.328	10.600	4.146	277
7	000	242.0	0.329	0.035	4.314	211
8	000	210.0	0.258	0.003	4.174	219
9	−−−	189.2	0.213	−0.022	3.992	145
10	++−	290.1	0.415	13.300	4.263	237
11	+−+	264.0	0.319	15.600	4.186	239

**Table 4:** Instability index of the formulations defined by factorial design.

Nanoemulsion	Instability Index	Profiles (RPM)
1	0.911	1000–4000
2	0.921	1000–4000
3	0.932	1000–4000
4	0.214	1000–4000
5	0.930	1000–4000
6	0.903	1000–4000
7	0.917	1000–4000
8	0.902	1000–4000
9	0.914	1000–4000
10	0.879	1000–4000
11	0.912	1000–4000

**Table 5:** Osmolality (mOsm/kg) of NEs monitored over a period of 60 days stored at 4 °C. The pH values were recorded on the day of production (day 0).

	DAY	0	1	3	7	14	28	60
S1	Osmo. mOsm/kg	162	155	153	154	153	153	153
	pH	4.230						
S2	Osmo. mOsm/kg.	157	150	155	152	152	152	153
	pH	4.382						
S3	Osmo. mOsm/kg.	156	157	150	152	150	152	146
	pH	4.163						
S4	Osmo. mOsm/kg.	164	155	153	161	156	154	152
	pH	4.011						
S5	Osmo. mOsm/kg	154	155	152	151	152	154	149
	pH	4.704						
S6	Osmo. mOsm/kg	151	153	146	142	144	151	152
	pH	4.359						
S7	Osmo. mOsm/kg.	154	156	145	144	146	152	151
	pH	4.307						
S8	Osmo. mOsm/kg	160	151	151	151	151	152	152
	pH	4.016						
S9	Osmo. mOsm/kg	160	158	153	154	157	158	157
	pH	4.176						
CTAB	Osmo. mOsm/kg	153	155	151	153	155	158	155
	pH	4.030						

**Table 6:** Encapsulation efficiency of Formulation F2, Formulation F7, CTAB-based NEs loaded with triamcinolone acetonide.

Formulation	F2	F7	CTAB-Based
Encapsulation Efficiency (%)	80.8	87.5	78.3

**Table 7:** Osmolality (mOsm/kg) of Formulation F2, Formulation F7, CTAB-based NEs loaded with triamcinolone acetonide over a period of 28 days.

Formulation	Day					
	0	1	3	7	14	28
F2	169	171	177	170	176	174
F7	138	142	146	141	144	149
CTAB-based	136	139	150	143	147	152

**Table 8:** Osmolality of the diluted formulation in different solution at different ratios.

Formulation	NEs in Sterile Saline Solution		NEs in Sterile Phosphate Buffer at Neutral pH	
	(1:1 Dilution)	(1:3 Dilution)	(1:1 Dilution)	(1:3 Dilution)
F2	204	243	479	601
F7	200	244	464	602
CTAB-based	236	261	481	606

## Data Availability

Not applicable.
